# Feeding Preferences of the Bean Leaf Beetle *(Ootheca* spp.) (Coleoptera: Chrysomelidae): Insights for Targeted Pest Control Strategies in Uganda

**DOI:** 10.3390/insects15070516

**Published:** 2024-07-10

**Authors:** Samuel Olaboro, Samuel Kyamanywa, Moses Lutaakome, Pamela Paparu, Charles Halerimana, Stanley Tamusange Nkalubo, Michael Hilary. Otim

**Affiliations:** 1 National Crops Resources Research Institute, Namulonge, Kampala P.O. Box 7084, Uganda; mosesluta5@gmail.com (M.L.); pamela.paparu@gmail.com (P.P.); tamusange@gmail.com (S.T.N.); 2 Department of Agricultural Production, College of Agricultural and Environmental Sciences, Makerere University, Kampala P.O. Box 7062, Uganda; skyamanywa@gmail.com (S.K.); chahalerimana@gmail.com (C.H.); 3 National Coffee Research Institute, Kituuza, Mukono P.O. Box 185, Uganda

**Keywords:** bean leaf beetles, *Ootheca* spp., abundance, foliar damage, host crops, feeding preference, *malakwang*, Malvaceae, Fabaceae

## Abstract

**Simple Summary:**

Bean leaf beetles (BLBs) are significant pests in Uganda, damaging crops such as beans and cowpeas, leading to substantial yield losses. They exhibit preferential feeding behaviour, targeting specific crops over others. Understanding these preferences can form the basis for identifying a potential trap crop that can be used to manage the pest at a low cost in a sustainable manner. A field study was conducted to determine the feeding preference of BLBs on various host crops among those commonly cultivated in Uganda, across different locations and seasons. This study was conducted in Arua and Lira districts in the first and second rainy seasons of 2018. Seven BLB host crops, i.e., common bean, cowpea, greengram, soybean, groundnuts, okra and roselle (locally known as *malakwang*), were selected for the study. The results showed that cowpea exhibited the highest abundance of BLBs among all crops and it had a high amount of foliar damage as well. Thus, it was selected as the most preferred host crop and can be recommended as a trap crop for managing *Ootheca* spp. With respect to locations and seasons, the pest was more abundant in Arua than in Lira and more abundant in 2018A than 2018B across all locations.

**Abstract:**

The bean leaf beetle (BLB) (*Ootheca* spp.) is a polyphagous pest causing significant yield losses in Uganda, particularly in the Northern and Eastern regions on various hosts plants. Despite its polyphagous behaviour, the BLB exhibits preferential feeding, offering an opportunity for targeted pest management. This study explored its feeding preferences across seven crops: common bean, cowpea, greengram, okra, roselle (*malakwang*), groundnuts, and soybean. This study was conducted in Arua and Lira districts using a randomized complete block design for two rainy seasons (2018A and 2018B). The results showed significant differences in BLB abundance and foliar damage among host crops, locations, days after planting and seasons. Cowpea was the most preferred crop while groundnuts was the least preferred. Therefore, cowpea can be recommended for use as a trap for managing *Ootheca* spp. in gardens where it is not the main crop. There was a higher pest abundance in Arua than in Lira. There was also a higher pest abundance in 2018A than in 2018B. These findings highlight the importance of understanding BLB’s feeding preferences for implementing effective IPM strategies, emphasizing the potential role of trap cropping, especially with cowpea, to minimize BLB damage in resource-constrained agricultural settings.

## 1. Introduction

The bean leaf beetles (BLBs) (*Ootheca* spp. [Coleoptera: Chrysomelidae]) are polyphagous pests of plants belonging to various families such as the Fabaceae family, which includes crops such as common bean (*Phaseolus vulgaris*) and cowpea (*Vigna unguiculata*) [1,2] and the Malvaceae family with crops such as okra (*Abelmoschus esculentus*) [3]. These pests are endemic to sub-Saharan Africa with thirteen species distributed in this area [4]. Four species of *Ootheca* beetles have been reported in Uganda: *O. mutabilis*, *O. proteus*, *O. orientalis* and *O. ugandae* [4,5]. In 2016–2017, the relative abundance of *O. mutabilis* and *O. proteus* were approximately 80.3% and 19.3%, respectively [5]. It was also observed that *Ootheca* beetles were more abundant in northern and eastern regions of Uganda where they extensively defoliated the common bean [6].

*Ootheca* spp. damage host crops in a variety of ways. The larvae feed on crop roots and nodules, causing yellowing of the leaves [7] and a reduction in the number of pods per plant [1]. Adult beetle leaf feeding is interveinal, making distinct round feeding holes [8], with a strong preference for young leaves [3]. Defoliation results in significant grain yield losses, which have been estimated at 18–31% in Tanzania [9] and 28.4–48.9% in Uganda [6] for common bean. Under high infestation, grain yield loss can reach 100% [10,11]. *Ootheca mutabilis* is also a confirmed vector of cowpea mosaic disease viruses [12].

Bean leaf beetle outbreaks normally manifest in the first growing season [13] and this leads to significant yield losses in the various host crops. Given the resource constraints faced by most farmers in Uganda, particularly financial constraints [14], effective management of such outbreaks are a challenge when they occur. This indicates a need to develop and promote management strategies that are relatively affordable and effective. Despite its polyphagous behaviour, bean leaf beetles have been observed to exhibit preferential feeding behaviour. This makes them cause more damage and losses to crops such as common bean and cowpea in comparison to other observed host crops [13,15]. Understanding the beetle’s feeding preferences can enable researchers to develop and provide farmers with pest control strategies that exploit this behaviour, particularly trap cropping, in order to minimise/prevent damage to the host crops of interest. It is important to note that although trap cropping also incurs a cost, i.e., land that would be used for growing food crops is instead used for the trap crops [13], it is still a cheaper and more eco-friendly control method than other methods such as chemical control. However, there is limited information regarding the feeding preferences of the bean leaf beetle among the most commonly grown crop hosts in Uganda. The purpose of this study was therefore to assess the feeding preferences of bean leaf beetles for different host crops. This study assessed bean leaf beetle abundance and foliar damage on different host crops and how these parameters are influenced by seasons, locations and seasonal crop growth.

## 2. Materials and Methods

### 2.1. Study Locations

This study was conducted in two agro-ecological zones, i.e., the Arua farmlands and Northern moist farmlands [16], specifically in the districts of Arua and Lira, respectively. These agro-ecological zones were selected because of their high infestation levels of bean leaf beetles [6]. Arua farmland is characterised by mean annual temperatures >20 °C, annual rainfall of 1000–1200 mm/year, following a unimodal rainfall pattern, with soils that are sandy in upland areas and dark clays in the lowland areas. The Northern moist farmlands are characterised by mean annual temperatures >20 °C, annual rainfall of 1200 mm/year, following a bi-modal rainfall pattern, with predominantly sandy soils with low organic matter and nutrient availability [16]. This study was conducted on farmer’s gardens and the criteria for selection of these gardens were:

- The gardens had to have had a bean leaf beetle host crop in the previous season. This was to ensure that there would be a good population of the *Ootheca* spp. teneral adults diapausing in the soil from the previous season that would eventually emerge and sufficiently infest the crops in this study.
- The garden had not been treated with any pesticides in the previous two seasons.

### 2.2. Host Crops Used

Seven host crops on which *Ootheca* spp. have been observed feeding were selected to be used in this study: common bean (*Phaseolus vulgaris*) variety Narobean 1, greengram (*Vigna radiata*) variety Narogram 1, groundnuts (*Arachis hypogaea*) var Serenut 13R, soybean (*Glycine max*) variety Maksoy 1N, cowpea (*Vigna unguiculata*) variety Secow 3W, roselle (*Hibiscus sabdariffa*) (henceforth known as *malakwang*) brown seeded local variety, and okra (*Abelmoschus esculentus*) variety Pusa Sawani. Each host crop was planted in individual plots that measured 4 m × 3 m. The recommended spacing for each crop was used, i.e., greengram (45 cm × 20 cm), common bean (50 cm × 10 cm), okra (50 cm × 30 cm), soybean (60 cm × 5 cm), cowpea (30 cm × 50 cm), groundnuts (50 cm × 10 cm) and *malakwang* (50 cm × 30 cm). This spacing caters to one seed per hill. The experiment was laid out in a randomized complete block design with four replicates. Upon establishment of the crops, weeding was conducted twice during the season at 4 and 8 weeks after planting. There was no fertiliser application or irrigation.

### 2.3. Study Layout

The study was conducted in the first (March to June) and second rains (September to December) of 2018, which from here on will be known as 2018A and 2018B, in the two districts. The planting dates were 28 March 2018 in Lira and 20 April 2018 in Arua for 2018A and 9 September 2018 and 11 September 2018 for 2018B in Lira and Arua, respectively.

### 2.4. Data Collection

Data were collected on the following parameters: bean leaf beetle abundance, flea beetle abundance and foliar damage. The abundance on the different host crops was determined in situ by counting adult beetles on 20 plants randomly selected per plot. Foliar damage was determined by scoring the mean percentage of the leaves injured using a visual rating scale of 0 to 5, where: 0 = no damage on the leaves, 1 = 1–5% damage, 2 = 6–25% damage, 3 = 26–50% damage, 4 = 51–75% damage and 5 = 76–100% damage [9,17] to each plant. Data collection was performed in the cool hours of 8:00 am–11:00 am when the pest was relatively inactive and would not readily fly away prior to observation. Data were collected weekly starting at 21 days after planting (DAP) for six weeks, i.e., 28, 35, 42, 49 and 52 DAP, respectively. Data on flea beetles’ abundance was collected because they were found feeding on the host crops in 2018B in a manner similar to that of bean leaf beetles. This suggested that their feeding contributed to the foliar damage evident on host crops in 2018B.

### 2.5. Data Analysis

Genstat 14th Edition computer package for Windows (https://genstat.kb.vsni.co.uk/) (accessed on 5 April 2024) and Microsoft Excel 2016 were used for data analysis. Prior to the analysis, the data were subjected to the Shapiro–Wilk test for normality and Bartlett’s test for homogeneity of variances, in Genstat. The *Ootheca* abundance was not normally distributed (the value was below the threshold of *p* < 0.05) and the variances were also not homogenised (the value was below the threshold of *p* < 0.05). The data were then transformed using the square root (x + 1.0) to homogenise the variances. The Shapiro–Wilk test was conducted on the transformed data, which did not significantly deviate from the data drawn from a beetle population with a normal distribution (W = 0.6236, probability: <0.001). The data were then subjected to analysis of variance (ANOVA) to assess the significance of differences in *Ootheca* abundance and foliar damage for the seasons, locations and days after planting. The effects of the seasons, locations, days after planting and host crops on *Ootheca* abundance and foliar damage were assessed using repeated measures analysis of variance (RMANOVA). In the RMANOVA, the season, location, days after planting and host crops constituted the treatment (fixed factors), while the replications were the blocks (random factors) [15]. A subsequent downstream analysis was conducted for each district using a general ANOVA to determine the seasonal variations in *Ootheca* abundance, with the foliar damage to the host crops and the growth stages in the seasons as factors.

For the treatments showing significant F-statistics, the means of abundance of both *Ootheca* and flea beetles and foliar damage for the host crops, locations, days after planting and seasons were separated using the least significant difference (LSD) test at a 5% probability level where there were significant differences.

## 3. Results

### 3.1. Influence of Host Crops, Locations and Seasons on Bean Leaf Beetle Abundance and Foliar Damage

The fixed effects of host crops, season and location significantly (*p* < 0.001) influenced the abundance of bean leaf beetles. The bean leaf beetles were significantly (df = 6, F = 11.53, *p* < 0.001) higher on cowpea than other host plants (Table 1). There was a significantly (df = 1, F = 36.20, *p* < 0.001) higher bean leaf beetle abundance in Arua than in Lira (Table 1). The bean leaf beetles were also significantly (df = 1, F = 281.11, *p* < 0.001) higher in 2018A than in 2018B (Table 1).

The foliar damage was significantly (df = 6, F = 85.69, *p* < 0.001) higher on *malakwang* followed by okra, cowpea, common bean, greengram, soybean and groundnuts, respectively (Table 1). With regard to location, the foliar damage was significantly (df = 1, F = 420.96, *p* < 0.001) higher in Arua than in Lira (Table 1).

### 3.2. Influence of Interactions of Host Crops, Locations and Seasons on Bean Leaf Beetle Abundance and Foliar Damage

There was a significant (df = 6, F = 3.60, *p* = 0.002) effect of host crops × seasons × locations on *Ootheca* abundance (Table S2). In Lira district, there was significance (df = 13, F = 9.92, *p* < 0.001) in the bean leaf beetle abundance on the host crops between the seasons. The bean leaf beetles were most abundant on *malakwang* in 2018A and there were no beetles observed in 2018B on any host crop (Table 2). In Arua district, the bean leaf beetle abundance was significant (df = 13, F = 12.72, *p* < 0.001) across seasons on the host crops. The beetles were most abundant on common beans in 2018A and least abundant on okra in 2018B (Table 2).

There was an equally significant effect (df = 6, F = 15.83, *p* < 0.001) of host crops × seasons × locations on foliar damage (Table S1). In Arua, the foliar damage was significant (df = 13, F = 15.93, *p* < 0.001) across seasons for the host crops (Table S3). The foliar damage observed on the host crops for both seasons in Arua was highest on cowpea in 2018A and least on groundnuts in 2018A (Table 2). The foliar damage in Lira was significant (df = 13, F = 44.62, *p* < 0.001) on the host crops for all seasons (Table S2). Among all of the host crops in both seasons in Lira, the foliar damage was highest on *malakwang* in 2018B and was not present on groundnuts in 2018B (Table 2).

### 3.3. Bean Leaf Beetle Abundance and Foliar Damage Progression across the Days after Planting in Different Seasons and Locations

Bean leaf abundance was significant for the interactions of; DAP × location (df = 5, F = 10.22, *p* < 0.001), DAP × season (df = 5, F = 19.70, *p* < 0.001), DAP × location × season (df = 5, F = 9.92, *p* < 0.001) and DAP × location × host crops × seasons (df = 30, F = 3.30, *p* < 0.001) (Table S1). In Arua district, bean leaf beetle abundance for the interaction of DAP × seasons × host crops was significant (df = 65, F = 3.51, *p* < 0.001) (Table S5). In 2018A, the common bean had the highest bean leaf beetle abundance at 35 DAP and the least was observed on okra, malakwang and groundnuts at 49 DAP (A). In 2018B, all of the crops apart from okra had their peak population at 21 DAP with common bean having the highest abundance (B). In Lira district, the bean leaf beetle abundance for the interaction of DAP × seasons × host crops was significant (df = 65, F = 3.51, *p* < 0.001) (Table S4). In 2018A, the highest bean leaf beetle population was observed on *malakwang* at 49 DAP and the least was on groundnuts, which had no beetles present on them except at 28 DAP. With the exception of common bean and groundnuts, which had their peak populations at 21 DAP and 28 DAP, respectively (C). The other host crops had their peak populations towards the end of the season (C). In 2018B, no bean leaf beetles were observed on any day after planting on the host crops (D).

The foliar damage was significant for the interactions of: DAP × locations (df = 5, F = 4.41, *p* < 0.001), DAP × seasons (df = 5, F = 4.97, *p* < 0.001) and DAP × host crops (df = 30, F = 3.38, *p* < 0.001) (Table S1).

In Arua district, there was significant (df = 65, F = 1.75, *p* < 0.001) foliar damage evident on the host crops across the successive DAP for both seasons (Table S5). In 2018A, cowpea had the highest peak abundance at 42 DAP and groundnuts had the least abundance at 35 DAP (E). In 2018B, the highest abundance was observed on common bean at 42 DAP and the least was on groundnuts at 28 DAP (F).

In Lira, there was also significant (df = 65, F = 1.75, *p* = 0.001) foliar damage evident on the host crops across the DAP for both seasons (Table S4). In 2018A, *malakwang* had the highest foliar damage at 56 DAP and the foliar damage significantly increased at the end of the season. Groundnut had the least foliar damage, at 21 DAP (G). In 2018B, *malakwang* had the highest foliar damage at 56 DAP and groundnut had no evident foliar damage at all DAP (H).

### 3.4. Influence of Host Crops on Abundance of Flea Beetles in 2018B

**Figure 1:**
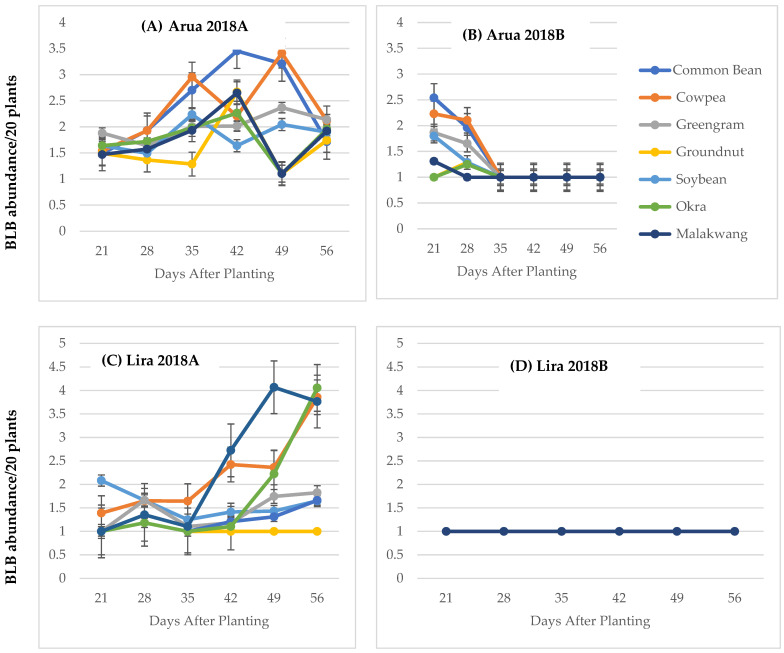

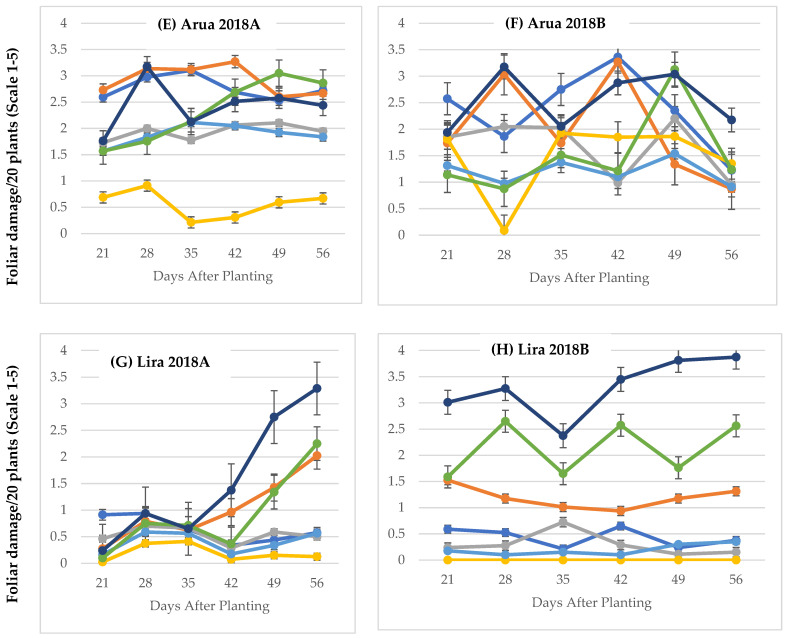
Progression of bean leaf beetle abundance (**A**–**D**) and foliar damage (**E**–**H**) on successive days after planting in 2018A and 2018B in Arua and Lira.

In 2018B, data were collected on flea beetle abundance because they were observed feeding on the host crops in a pattern similar to *Ootheca* beetles. Data on their abundance could be used to explain the trends in foliar damage on the host crops. The impact of host crops on flea beetle abundance was significant (df = 6, F = 60, *p* < 0.001) in 2018B. These beetles were most abundant on *malakwang* and okra and occurred in lower numbers on greengram (Table 3). The foliar damage on the host crops was also significant (*p* < 0.001). *Malakwang* had the highest damage, followed in decreasing order by okra, cowpea and common bean (Table 3). In 2018B, no bean leaf beetles were observed in Lira (**D**) and they were only observed at 21 and 28 DAP in Arua (Figure 1B).

## 4. Discussion

The purpose of this study was to assess the abundance and foliar damage attributed to *Ootheca* beetles on different host plants as influenced by location, season and seasonal crop growth. The results obtained indicated that there were significant differences in abundance and foliar damage of *Ootheca* beetles on the host crops. Cowpea had the highest overall *Ootheca* abundance followed by *malakwang*, common bean, okra, greengram, soybean and groundnuts, respectively. *Malakwang* had the highest foliar damage followed by okra, cowpea, common bean, greengram, soybean and groundnuts, respectively. *Ootheca* beetle abundance and foliar damage were significantly higher in Arua compared to Lira, and for both locations the *Ootheca* beetles were more abundant in 2018A than 2018B. There were no *Ootheca* beetles observed in Lira in 2018B, while in Arua, they were observed in the first two successive DAP before they were no longer observed. The *Ootheca* beetles were more abundant on the host crops from the Fabaceae family than the Malvaceae family. However, crops in the Malvaceae family had higher foliar damage, particularly in 2018B, and this foliar damage may have been significantly contributed to by flea beetles. Flea beetles (*Nisotra* sp.) were observed feeding in a similar pattern to the *Ootheca* beetles and these contributed to the foliar damage observed particularly in 2018B in both locations.

*Ootheca* beetles were most abundant on cowpea, probably because both the pest [4] and the host [18,19,20,21] are indigenous to Africa and are thus suspected to have co-evolved together. This could explain why *Ootheca* spp. preferred cowpea to other host crops. In fact, *O. mutabilis,* the most abundant species of *Ootheca* in Uganda, is referred to as the cowpea beetle in recognition of its preference for cowpea [20]. Nutritive content may also be another reason for *Ootheca* beetles’ preference for cowpea. It was reported that proteins and carbohydrates were the most important macro-nutrients for herbivorous insects [21]. Studies show that cowpea leaves have up to 34.91% protein and 31.11% carbohydrate [22], common bean leaves have up to 24.5% protein and 19.2% carbohydrate, and *malakwang* leaves has up to 17.27% protein and 48.33% carbohydrate [23]. A similar study of the feeding preferences of the polyphagous forest tent caterpillar (*Malacosoma disstria* Hübner) showed a preference for diets with a composition ratio that was biased towards protein [21]. Studies by [24] on mustard leaf beetles (*Phaedon cochleariae*) and [25] on mealworm beetles (*Tenebrio molitor*) showed that polyphagous herbivorous beetles selected crop hosts based on foliage having a higher protein content than carbohydrates in order to achieve high fitness. The *Ootheca* beetle, being a polyphagous pest, might display similar dietary preferences, and among the host crop leaves mentioned, cowpea has the protein:carbohydrate ratio that fits the herbivore-favoured parameters reported by [21]. Nevertheless, further studies may be required to test this hypothesis. The preference of *Ootheca* spp. for cowpea are also corroborated by the work of [15], who found that *Ootheca* adults and below ground life stages were more abundant on a cowpea crop than on a common bean crop.

It is also noteworthy that the protein content of *malakwang* leaves (up to 17.27%) is lower than that of soybean (up to 22.9%) [26], greengram (up to 26.71%) [27], groundnuts (up to 18%) [28] and okra (up to 21.55%) [29]. This would imply that these crops would be preferred to *malakwang*; however, this was not the case. This may be due to the presence of pronounced trichomes, particularly on soybean [30] and greengram [31], that may have had a limiting effect on feeding by the *Ootheca* beetles since they provide physical resistance to crop pests [32,33]. This assertion is supported by [34], who noted that a substantial presence of trichomes affected the feeding by multiple chrysomelid beetles. Furthermore, it was observed by [35] that the Mexican leaf beetle (*Cerotoma trifurcata*), also referred to as a bean leaf beetle, significantly preferred to feed on soybean varieties with fewer trichomes. The *Ootheca* beetles may have similarly focused more on *malakwang* than other host crops because it did not have this physical barrier. Similar to this study, a low population of *Ootheca* beetles was also observed on soybean by [15]. This is further supported by the work of [36,37], who observed minimal beetle emergence on fields that had soybean.

The disparity in *Ootheca* beetle abundance and foliar damage between Arua and Lira may be attributed to differences in the climatic conditions of the two locations. Lira experiences a bimodal rainfall distribution, while Arua experiences a unimodal rainfall distribution [16]. A reduction in beetle abundance as the season progressed and rains intensified was also noted by [38]. This may explain why the beetle abundance on the host crops in Lira increased as the first season came to a close, while it declined in Arua, where the rains were becoming more intense as the season progressed. Furthermore, the trial fields in Lira were prepared using ox-ploughs while those in Arua were prepared using hand-hoes. The ox-ploughs dig deeper than the hand-hoes, and as a result, they may have unearthed more infantile *Ootheca* beetles, thus leaving them vulnerable to desiccation by the sun. This supports the observation by [7] that ploughing unearths infantile *Ootheca* beetles that dwell in the soil. This could have reduced the population present in the soil prior to planting in Lira. This may have contributed to Arua generally having more beetles than Lira.

The *Ootheca* beetle abundance in both locations was higher in the 2018A compared to 2018B. No *Ootheca* spp. were present in 2018B in Lira. This reaffirms earlier studies by [13,38] that the *Ootheca* beetle is primarily a first season pest. The appearance of *Ootheca* beetles on host crops in the first weeks of 2018B in Arua may be because farmers in Arua delay planting of legumes in the first season [5]. This ensured that the beetles had a food source that sustained them into the second season and so they were present and able to infest the trial when it was established. It was reported by [15] that the *Ootheca* beetles observed in the second season were a carry-over from the previous season’s generation. All of the host crops in both locations experienced a steep decline in the abundance of *Ootheca* beetles from 2018A to 2018B. All crops, with the exception of *malakwang* and okra, experienced a corresponding decline in foliar damage in 2018B cross the two seasons.

Flea beetles in 2018B were observed feeding on okra and *malakwang* in a pattern that was similar to that of the *Ootheca* beetles. Flea beetles have been confirmed as pests of both okra [39,40] and *malakwang* [41,42]. The higher foliar damage observed on *malakwang* and okra can be attributed to flea beetles and the lesser damage on the other host crops in 2018B may be attributed to the few remaining *Ootheca.* This indicates that foliar damage is not entirely reliable as an indicator of exclusive *Ootheca* beetle feeding. A separate study may be required to quantify the amount of foliar damage that each of these two pests cause individually to these host crops.

The *Ootheca* beetle abundance/population distribution across the successive DAP for all of the crops in both locations had at least two peaks over the course of the season. In Lira district, there was a general increase in *Ootheca* beetle abundance from 35 DAP onwards on all of the host crops. This is consistent with the observation by [38] that *Ootheca* beetle abundance increased towards the end of the season in the northern moist farmlands AEZ, particularly in the first season. This increase was significant in cowpea, *malakwang* and okra. This may be because these crops have longer life spans than the rest, and as a result, the vegetative and flowering stages were ongoing for them while the other crops were undergoing later growth stages/senescence [37,43]. As a result, the *Ootheca* beetles migrated to these three crops in order to continue feeding on the fresher foliage and flowers. *Ootheca* abundance in Arua generally decreased towards the end of the data collection process. This supports the report by [44], who observed a drop in *Ootheca* abundance on common bean towards the end of the growing season. Rainfall in 2018 was higher in Arua (1439.2 mm) than in Lira (1347.3 mm) [15], and ref. [45] noted that heavy rainfall reduced the abundance of *Ootheca bennigseni*. As a result, the reduction in *Ootheca* abundance in Arua towards the end of 2018A might have been due to the higher rainfall experienced in comparison to Lira. A study conducted alongside this study by [15] showed that rainfall in Lira was higher earlier in the season. This could have led to increased mortality of the beetles by dislodging them from leaves and subsequently drowning them. However, as the rain reduced across 2018A, the beetle abundance increased. This, particularly in Lira, may have limited the feeding of the pest early in the season when the pest had been observed by [9,13,43] to inflict the most damage.

## 5. Conclusions

Cowpea had the highest overall *Ootheca* beetle abundance among all of the host crops. The population of *Ootheca* beetles was higher in the first season than in the second season. The *Ootheca* beetles were present in Arua farmlands AEZ (Arua) in both seasons; however, they were present in northern moist farmlands (Lira) only in the first season. In the Arua farmlands AEZ, the *Ootheca* beetles were only present in the beginning of the season and were not observed after that. *Ootheca* beetles were most abundant on the host crops from early in the growth season to the middle of the season. Flea beetles significantly increased the foliar damage on the host crops, particularly okra and *malakwang*, and as a result, the foliar damage present on them could not solely be attributed to *Ootheca* beetles as in the case of the other host crops.

Cowpea should be studied further to determine its effectiveness as a trap crop for the control of BLBs. Since cowpea belongs to the same family as a number of the other host crops, future studies should also investigate if it may attract other pests that may infest/infect the primary crop, thereby making its use as a trap crop a liability rather than an asset. Furthermore, the economic ramifications of a farmer setting aside land to establish a trap crop rather than the crop of interest should also be determined in order to justify the use of the trap crop. Management of BLBs should be emphasised in the first season to limit foliar damage caused by the pest. Though labelled previously as a seedling pest, this pest was present throughout the growth season following an outbreak so its management should be continued for most of the season.

## Figures and Tables

**Table 1:** Abundance of bean leaf beetles and foliar damage as influenced by host crops, locations, and seasons.

Fixed Factors	Bean Leaf Beetle Abundance/20 Plants (Mean ± SE)	Foliar Damage (Mean ± SE)
Host Crops		
Common bean	1.60 ± 0.92 ^bc^	1.55 ± 1.17 ^cd^
Cowpea	1.74 ± 1.04 ^c^	1.72 ± 1.14 ^d^
Greengram	1.42 ± 0.60 ^abc^	1.11 ± 0.97 ^bc^
Groundnuts	1.17 ± 0.43 ^a^	0.56 ± 0.89 ^a^
Malakwang	1.54 ± 1.07 ^bc^	2.41 ± 1.13 ^e^
Okra	1.40 ± 0.80 ^ab^	1.73 ± 1.01 ^d^
Soybean	1.31 ± 0.56 ^ab^	0.92 ± 0.80 ^ab^
Location		
Arua	1.58 ± 0.77 ^b^	1.94 ± 1.00 ^b^
Lira	1.33 ± 0.85 ^a^	0.913 ± 1.10 ^a^
Season		
2018A	1.81 ± 1.00 ^b^	1.43 ± 1.10 ^a^
2018B	1.20 ± 0.32 ^a^	1.42 ± 1.24 ^a^

Means bearing different letters within columns are significantly different at *p* < 0.05 based on Tukey’s HSD. Values are means estimated using the model (±standard errors).

**Table 2:** Bean leaf beetle abundance and foliar damage on host crops within locations for both seasons.

Host Crops	Adult Bean Leaf Beetle Abundance/20 Plants (Mean ± SE)				Foliar Damage/20 Plants (Mean ± SE)			
	Arua		Lira		Arua		Lira	
	2018A	2018B	2018A	2018B	2018A	2018B	2018A	2018B
Common Bean	2.42 ± 1.25 ^f^	1.42 ± 0.65 ^abcd^	1.58 ± 0.62 ^abc^	1.00 ± 0.00 ^a^	2.88 ± 0.45 ^f^	2.24 ± 0.85 ^cdef^	0.64 ± 0.36 ^abcd^	0.43 ± 0.30 ^abc^
Cowpea	2.34 ± 0.94 ^ef^	1.39 ± 0.58 ^abcd^	2.22 ± 1.39 ^cd^	1.00 ± 0.00 ^a^	2.92 ± 0.33 ^f^	1.73 ± 1.25 ^bcd^	1.02 ± 1.09 ^cde^	1.20 ± 0.39 ^de^
Greengram	2.01 ± 0.63 ^def^	1.25 ± 0.38 ^abc^	1.42 ± 0.61 ^ab^	1.00 ± 0.00 ^a^	1.94 ± 0.36 ^bcde^	1.68 ± 1.16 ^bcd^	0.53 ± 0.31 ^abcd^	0.30 ± 0.49 ^ab^
Groundnuts	1.61 ± 0.65 ^abcd^	1.05 ± 0.17 ^a^	1.03 ± 0.15 ^ab^	1.00 ± 0.00 ^a^	0.56 ± 0.43 ^a^	1.48 ± 1.30 ^bc^	0.19 ± 0.19 ^a^	0.00 ± 0.00 ^a^
Malakwang	1.78 ± 0.67 ^bcde^	1.05 ± 0.25 ^a^	2.34 ± 1.72 ^d^	1.00 ± 0.00 ^a^	2.43 ± 0.37 ^cdef^	2.54 ± 0.94 ^ef^	1.54 ± 1.46 ^ef^	3.30 ± 0.70 ^g^
Okra	1.78 ± 0.56 ^bcde^	1.04 ± 0.20 ^a^	1.76 ± 1.31 ^bcd^	1.00 ± 0.00 ^a^	2.34 ± 0.61 ^def^	1.52 ± 0.94 ^bc^	0.92 ± 0.82 ^bcde^	2.13 ± 1.03 ^f^
Soybean	1.83 ± 0.78 ^cdef^	1.18 ± 0.33 ^ab^	1.23 ± 0.41 ^ab^	1.00 ± 0.00 ^a^	1.89 ± 0.29 ^bcde^	1.20 ± 0.75 ^ab^	0.40 ± 0.27 ^abc^	0.20 ± 0.21 ^a^

Means bearing different letters within columns are significantly different at *p* < 0.05 based on Tukey’s HSD. Values are means estimated using the model (±standard errors).

**Table 3:** Effect of host crops on flea beetle abundance and foliar damage observed on the host crops in 2018B.

Host Crop	Flea Beetle Abundance/20 Plants	Foliar Damage
Common bean	1.00 ± 0.00 ^a^	1.34 ± 0.16 ^bc^
Cowpea	1.00 ± 0.00 ^a^	1.46 ± 0.14 ^bc^
Greengram	1.03 ± 0.02 ^a^	0.99 ± 0.16 ^ab^
Groundnuts	1.00 ± 0.00 ^a^	0.74 ± 0.17 ^a^
Malakwang	2.29 ± 0.16 ^b^	2.92 ± 0.13 ^d^
Okra	2.13 ± 0.13 ^b^	1.82 ± 0.15 ^c^
Soybean	1.00 ± 0.00 ^a^	0.70 ± 0.11 ^a^

Values bearing different letters within columns are significantly different at *p* < 0.05 based on Tukey’s HSD. Values are means estimated using the model (±standard errors).

## Data Availability

All data are provided in the main body of the published article and Supplementary Materials.

## Supplementary Materials

The following supporting information can be downloaded at: https://www.mdpi.com/article/10.3390/insects15070516/s1, Figure S1: Rainfall and temperature distribution against weeks for 2018A and 2018B (used with permission from co-author Lutaakome Moses). Table S1: Analysis of Variance for Bean leaf Beetle abundance and foliar damage. Table S2: ANOVA for Bean leaf beetle abundance and foliar damage for Lira with treatment nested in seasons. Table S3: ANOVA for Bean leaf beetle abundance and foliar damage for Arua with treatment nested in seasons. Table S4: ANOVA for Bean leaf beetle abundance and foliar damage for Lira with treatment and days after planting nested in seasons. Table S5: ANOVA for Bean leaf beetle abundance and foliar damage for Arua with treatment and days after planting nested in seasons.
