# Supplementary material for: Feeding Preferences of the Bean Leaf Beetle (Ootheca spp.) (Coleoptera: Chrysomelidae): Insights for Targeted Pest Control Strategies in Uganda
Source: Insects. 2024 Jul 10;15(7):516. doi: 10.3390/insects15070516 (PMC11277491; doi:10.3390/insects15070516)
Supplement: Supplementary file 1 [file insects-15-00516-s001.zip › insects-2937145-supplementary.pdf]

Table S1: Analysis of Variance for Bean leaf Beetle abundance and foliar damage

**Source of variation**	**Bean leaf beetle abundance**					**Foliar damage**			
	**Df**	**Sums of squares**	**Mean square**	**Variance ratio (F)**	**F Prob.**	**Sums of squares**	**Mean squares**	**Variance ratio**	**F Prob.**
**Replication stratum**	3	2.7369	0.9123	3.01		3.2452	1.0817	2.57	
**Host Crop**	6	0.9703	3.4951	11.53	<.001	216.7101	36.1184	85.69	<.001
**Season**	1	5.1899	85.1899	281.11	<.001	0.0060	0.0060	0.01	0.905
**Location**	1	0.9696	10.9696	36.20	<.001	177.4287	177.4287	420.96	<.001
**Days After Planting**	5	6.6071	1.3214	4.36	<.001	9.4476	1.8895	4.48	<.001
**Location x Season**	1	0.5704	0.5704	1.88	0.171	18.9007	18.9007	44.84	<.001
**Location x Host Crop**	6	1.9568	1.9928	6.58	<.001	65.3052	10.8842	25.82	<.001
**Season x Host Crop**	6	2.4847	2.0808	6.87	<.001	46.0371	7.6728	18.20	<.001
**Host Crop x Days After Planting**	30	7.9129	0.5971	1.97	0.002	42.6836	1.4228	3.38	<.001
**Location x Days After Planting**	5	5.4880	3.0976	10.22	<.001	13.6212	2.7242	6.46	<.001
**Season x Days After Planting**	5	29.8514	5.9703	19.70	<.001	10.4799	2.0960	4.97	<.001
**Host Crop x District x Days After Planting**	30	19.7477	0.6583	2.17	<.001	25.3395	0.8446	2.00	0.001
**Host Crop x Season x Days After Planting**	30	18.1392	0.6046	2.00	0.002	15.5125	0.5171	1.23	0.192
**Location x Season x Days After Planting**	5	15.0350	3.0070	9.92	<.001	5.2809	1.0562	2.51	0.030
**Location x Host Crop x Season**	6	6.5398	1.0900	3.60	0.002	40.0296	6.6716	15.83	<.001
**Host Crop x Location x Season x Days After Planting**	30	29.9567	0.9986	3.30	<.001	14.3026	0.4768	1.13	0.291
**Residuals**	501	151.8286		0.3031		501	211.1629		0.4215
**Total**	671	455.9850			671	915.4933			

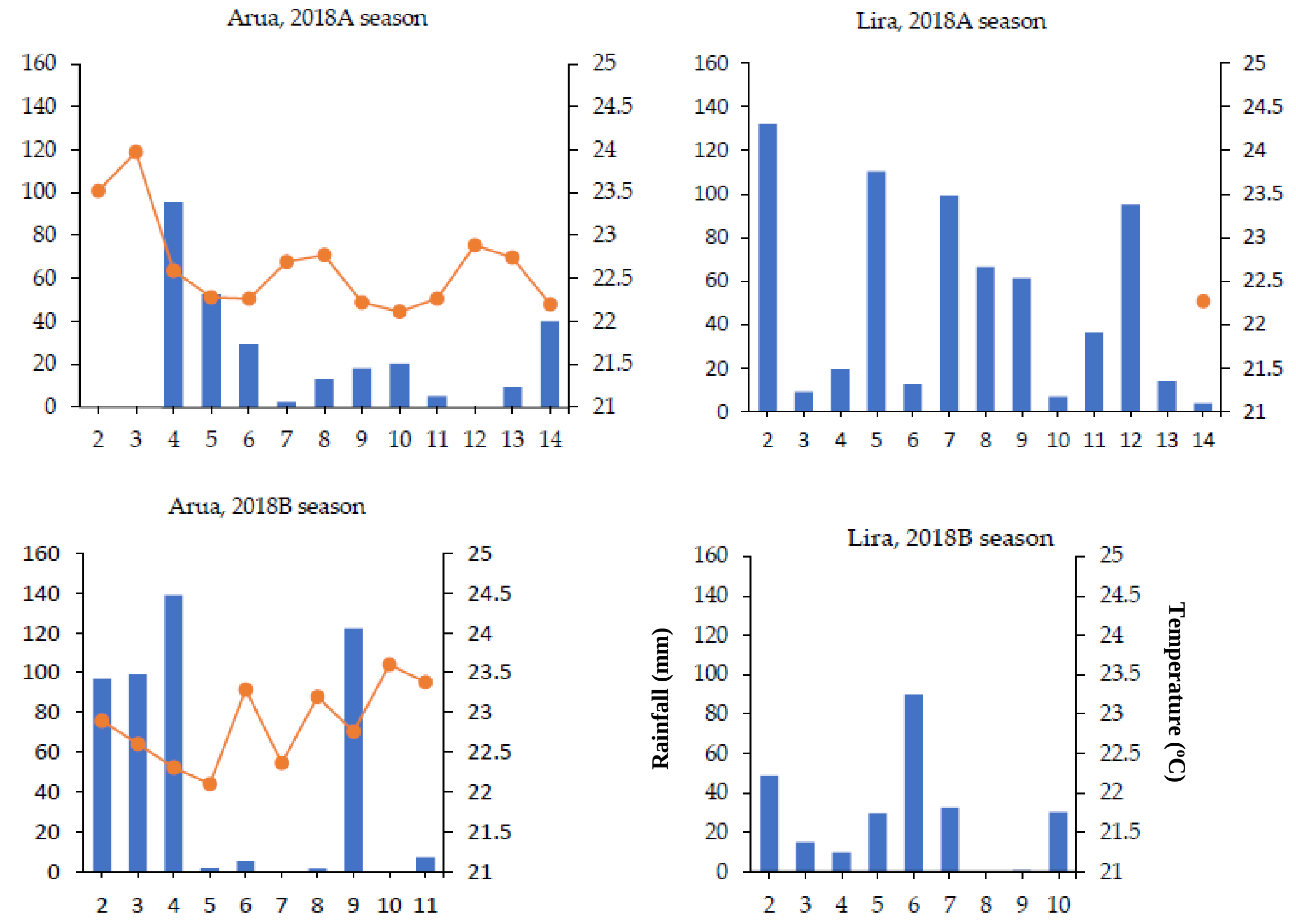

Figure S1: Rainfall and temperature distribution against weeks for 2018A and 2018B (used with permission from co-author Lutaakome Moses).

Table S2: ANOVA for Bean leaf beetle abundance and foliar damage for Lira with treatment nested in seasons

Source of variation	Bean leaf beetle abundance					Foliar damage			
	Df	Sums of squares	Mean square	Variance ratio (F)	F Prob.	Sums of squares	Mean squares	Variance ratio	F Prob.
**Replication stratum**	3	0.4902	0.1634	0.30		6.7349	2.2450	5.11	
**Season .Treatment**	13	0.2180	5.4014	9.92	<.001	254.9191	19.6092	44.62	<.001
**Residual**	319	173.6952	0.5445			140.1946	0.4395		
**Total**	335	244.4034				401.8486			

Table S3: ANOVA for Bean leaf beetle abundance and foliar damage for Arua with treatment nested in seasons

Source of variation	Bean leaf beetle abundance					Foliar damage			
	Df	Sums of squares	Mean square	Variance ratio (F)	F Prob.	Sums of squares	Mean squares	Variance ratio	F Prob.
**Replication stratum**	3	2.8773	0.9591	2.35		0.7301	0.2434	0.38	
**Season .Treatment**	13	67.4938	5.1918	12.72	<.001	132.0696	10.1592	15.93	<.001
**Residual**	319	130.2408	0.4083			203.4163	0.6377		
**Total**	335	200.6120				336.2161			

Table S4: ANOVA for Bean leaf beetle abundance and foliar damage for Lira with treatment and days after planting nested in seasons

Source of variation	Bean leaf beetle abundance					Foliar damage			
	Df	Sums of squares	Mean square	Variance ratio (F)	F Prob.	Sums of squares	Mean squares	Variance ratio	F Prob.
**Replication stratum**	3	0.4902	0.1634	0.48		6.7349	2.2450	7.59	
**DAP**	5	0.0873	4.0175	11.87	<.001	13.9452	2.7890	9.43	<.001
**Season .Treatment**	13	70.2180	5.4014	15.96	<.001	254.9191	19.6092	66.31	<.001
**Season.Treatment.DAP**	65	69.3536	1.0670	3.15	<.001	52.6174	0.8095	2.74	<.001
**Residual**	249	84.2543	0.3384			73.6320	0.2957		

**Total**	335	244.4034				401.8486			

Table S5: ANOVA for Bean leaf beetle abundance and foliar damage for Arua with treatment and days after planting nested in seasons

**Source of variation**	**Bean leaf beetle abundance**					**Foliar damage**			
	**Df**	**Sums of squares**	**Mean square**	**Variance ratio (F)**	**F Prob.**	**Sums of squares**	**Mean squares**	**Variance ratio**	**F Prob.**
**Replication stratum**	3	2.8773	0.9591	3.57		0.7301	0.2434	0.45	
**DAP**	5	2.0079	0.4016	1.49	0.192	9.1236	1.8247	3.41	0.005
**Season .Treatment**	13	67.4938	5.1918	19.31	<.001	132.0696	10.1592	18.98	<.001
**Season.Treatment.DAP**	65	61.2893	0.9429	3.51	<.001	60.9816	0.9382	1.75	0.001
**Residual**	249	66.9436	0.2688			133.3111	0.5354		
**Total**	335	200.6120				336.2161			
